# Glycosylation of ALV-J Envelope Protein at Sites 17 and 193 Is Pivotal in the Virus Infection

**DOI:** 10.1128/jvi.01549-21

**Published:** 2022-02-23

**Authors:** Moru Xu, Kun Qian, Hongxia Shao, Yongxiu Yao, Venugopal Nair, Jianqiang Ye, Aijian Qin

**Affiliations:** a Ministry of Education Key Lab for Avian Preventive Medicine, Yangzhou University, Yangzhou, Jiangsu, People’s Republic of China; b Jiangsu Co-innovation Center for Prevention and Control of Important Animal Infectious Diseases and Zoonoses, Yangzhou, Jiangsu, People’s Republic of China; c The Pirbright Institute & UK-China Centre of Excellence on Avian Disease Research, Pirbright, Surrey, United Kingdom; Ulm University Medical Center

**Keywords:** ALV-J, envelope protein, function analysis, glycosylation

## Abstract

Glycans on envelope glycoprotein (Env) of the subgroup J avian leukosis virus (ALV-J) play an essential role in the virion integrity and infection process. In this study, we found that, among the 13 predicted N-linked glycosylation sites (NGSs) in *gp85* of Tibetan chicken strain TBC-J6, N17, and N193/N191 are pivotal for virus replication. Further research illustrated that a mutation at N193 weakened Env-receptor binding in a blocking assay of the viral entrance, coimmunoprecipitation, and ELISA. Our studies also showed that N17 was involved in Env protein processing and later virion incorporation based on the detection of p27 and Env protein in the supernatant and *gp37* in the cell culture. This report is systematic research on clarifying the biological function of NGSs on ALV-J *gp85*, which would provide valuable insight into the role of *gp85* in the ALV life cycle and anti-ALV-J strategies.

**IMPORTANCE** ALV-J is a retrovirus that can cause multiple types of tumors in chickens. Among all the viral proteins, the heavily glycosylated envelope protein is especially crucial. Glycosylation plays a major role in Env protein function, including protein processing, receptor attachment, and immune evasion. Notably, viruses isolated recently seem to lose their 6^th^ and 11^th^ NGS, which proved to be important in receptor binding. In our study, the 1^st^ (N17) and 8^th^ (N193) NGS of *gp85* of the strain TBC-J6 can largely influence the titer of this virus. Deglycosylation at N193 weakened Env-receptor binding while mutation at N17 influenced Env protein processing. This study systemically analyzed the function of NGSs in ALV-J in different aspects, which may help us to understand the life cycle of ALV-J and provide antiviral targets for the control of ALV-J.

## INTRODUCTION

Avian leukosis virus subgroup J (ALV-J) belongs to the family Retroviridae and is associated with multiple types of hematopoietic tumors in chickens. ALV-J differs from the other 9 subgroups of ALV based on the identity of the envelope protein, host range, viral interference, and cross-neutralization patterns (1). Since ALV-J was first isolated from broiler chicken in the UK in 1988, it has been reported all over the world (2). During the early 2000s, ALV-J was found subsequently in broiler chicken and layer chicken in China (3, 4). With the help of the eradication program, ALV-J occurrence in broiler and layer chickens has significantly decreased since 2010. Meanwhile, local Chinese chickens are subjected to consistent ALV-J infection (5). Recently, analysis of the re-emerging outbreak of ALV-J in China indicated that there are new strains with the same ancestors, but they had gone through different evolutionary trajectories (6). Those new isolated ALV-J strains show salient differences in clinical symptoms compared with those from the early 2000s, including morbidity, mortality, and organ type of tumors. The sequence conservation of *gp85* of the newly isolated strains was only 78.5% to 95.6% compared with the ALV-J prototype strain HPRS-103 (7). Molecular epidemiology results showed that ALV-J strains are clustered into several independent branches. One interesting fact is that those branches have a high dependency on the host genetic background and geographic location (7, 8).

It is well known that the glycosylation of retroviral envelope proteins exerts great importance on the fitness of the virus. The “glycan shield” masks the surface of the Env protein and occupies over 40% of the total weight. When the *env* gene is translated, the first 60 amino acids serve as the signal peptide to help the newly formed peptide to enter the endoplasmic reticulum (ER) where it undergoes further glycosylation, forms disulfide bonds, folds, and oligomerizes. This precursor is then transported to the Golgi apparatus where it is cleaved by cellular proteases, and *gp85*-*gp37* is finally formed (9). Only the proper modified protein can reach the cell membrane and be incorporated into viral particles, while the poorly glycosylated or misfolded proteins cannot fulfill this biological function during the budding of the virion at the cell surface. The importance of glycans in Env protein is likely to extend beyond protein modification and folding, and they can serve as the stabilizer of the protein (10, 11) and play a pivotal role in viral receptor binding (12). Through glycosylation, the surface structure can be altered dramatically, finally influencing the protein-protein interactions. In addition, previous reports demonstrated the involvement of N-linked glycosylation in immune evasion of some viruses (13, 14).

ALV-J virus enters the cells by binding with Na^+^/H^+^ exchanger type 1 (NHE1) (15), which determines its host range. The 28 to 39 amino acids of the first extracellular loop (ECL1) of NHE1 play a critical role in binding to gp85 (16, 17), among which W38 is especially pivotal. A study on HPRS103 has demonstrated that the amino acids 38 to 131 and 159 to 283 of *gp85* serve as the NHE1-binding domain assisting virus invasion (18). In addition, the 6^th^ and 11^th^ NGS are indispensable.

However, more and more field cases that were contradictory to previous observations have since appeared. The reports had claimed to isolate ALV-J from some rare breeds of chicken and diverse avian hosts, including quails, ducks, and gray partridge (19–21), which were originally thought to be resistant to ALV-J infection. Some of them had NHE1 with mismatching W38 to susceptibility/resistance that was previously identified but still conferred susceptibility to ALV-J infection.

Several recent epidemiological studies concluded that ALV-J is mutating and evolving at a rather fast speed (5, 9). These observations together with the special cases from wild birds prompted us to hypothesize that ALV-J strains from different origins may make use of different receptor-binding domains and NGSs to enter cells. If our hypothesis on the potential additional role of NGS is correct, the rapid evolutionary trend observed in ALV-J can be a threat to the current antiviral strategies.

In this study, we used the ALV-J strain TBC-J6 (22) to examine the role of NGS in the Env glycoprotein in virus infectivity and fitness. Using targeted mutagenesis of infectious clones, N17 and N193 had been identified as two key N-linked glycosylation sites that can influence virus titer significantly. We found that N17 was pivotal in the Env protein processing and later virion incorporation while N193 was involved in receptor binding.

## RESULTS

### Identification of the NGSs of different ALV strains.

**FIG 1:**
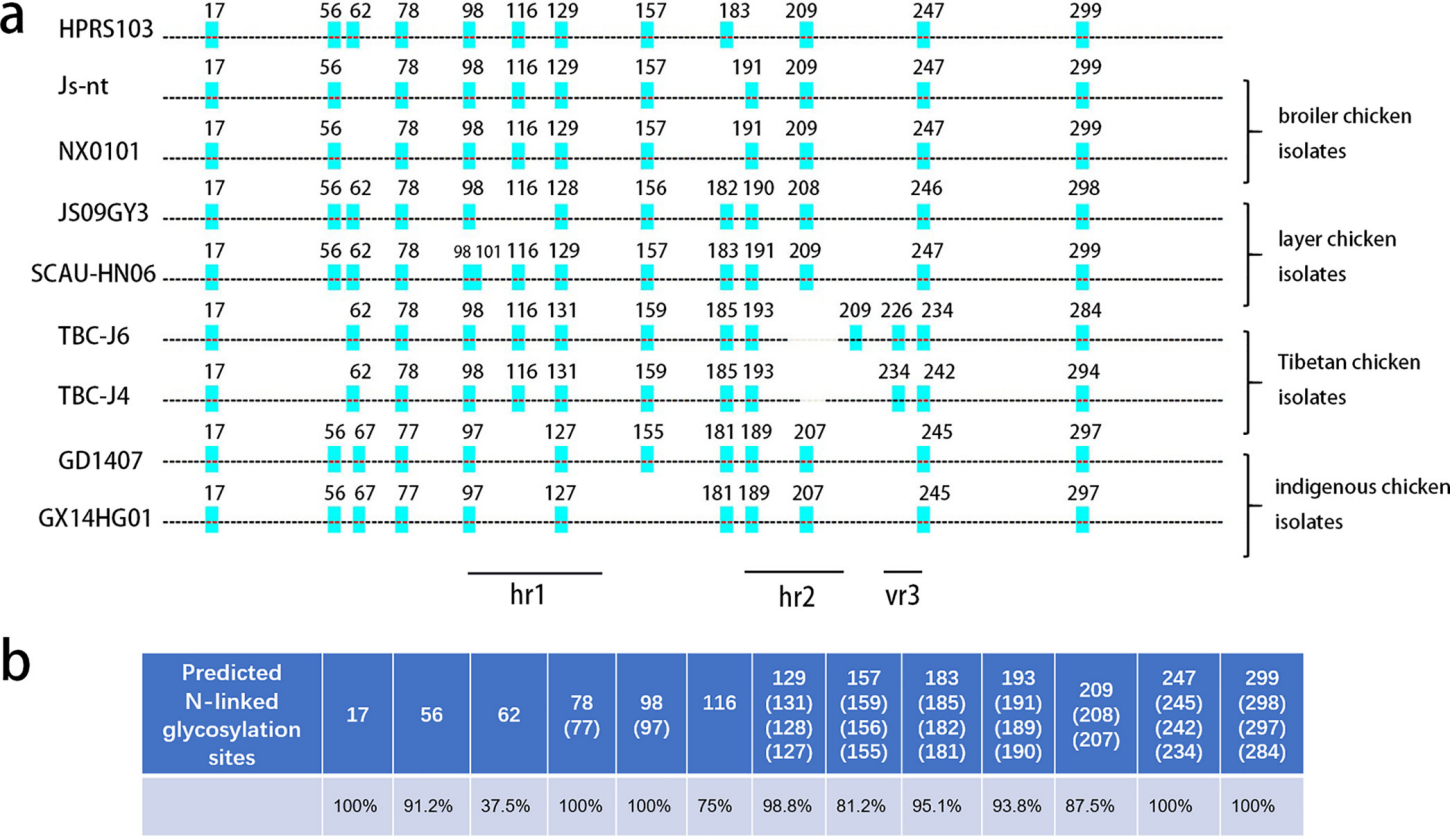
Analysis of NGSs in *gp85* of different ALV-J strains. (a) Maps of the *gp85* domains of different ALV-J strains. Alignment of *gp85* sequences of ALV-J based on HPRS103 (Z46390) from GenBank and comparison of their potential NGSs. Broiler chicken isolates JS-nt (HM235667), NX0101 (AY897227); layer chicken isolates JS09GY3 (GU982308) & SCAU-HN06 (KQ900844); indigenous chicken isolates GD1407 (KU500034) & GX14HG01 (KU997685); Tibetan chicken isolates TBC-J4 (MT409624) and TBC-J6 (MT409625) were analyzed. Potential NGSs are marked according to their position in *gp85*. Hr1 and hr2 are also indicated. (b) Percentage of the presence of different predicted NGSs in 80 ALV-J strains. Numbering is based on the HPRS103 sequence.

We chose 8 ALV-J strains with different background information and mapped each of the predicted NGSs using an online program. According to the program, a score of 0.5 was set as the cutoff line with a score above 0.5 being positive and a score below 0.5 being negative for glycosylation. As shown in Fig. 1A, 11 to 14 NGSs per *gp85* monomer of Env were present from different strains. They were numbered according to their positions. Based on the HPRS103 sequence, the disappearance of some NGSs was due to either losing the basic motif of N-X-S/T (X≠P) or the predicted scores were below the threshold of 0.5. When the predicted score was under 0.5, it means the site is less likely to be glycosylated. Fig. 1A shows that most predicted NGSs in gp85 of ALV-J were conserved, but different strains either gain or lose some NGSs compared to prototype strain HPRS103. In addition, isolates from the same chicken flocks (i.e., layer chicken flocks, broiler chicken flocks, and indigenous chicken flocks) had nearly the same NGSs. Tibetan chicken isolates TBC-J4 and TBC-J6 had unique NGSs between hypervariable region 2 (hr2) and variable region 3 (vr3), which were different from all other strains. The corresponding variable regions were also marked.

To confirm our assumption that most NGSs were not essential in different strains, we mapped and predicted NGSs in 80 randomly selected ALV-J strains from GenBank (Table S1). The ALV-J strains from wild birds, gray partridges, and ducks were also included but showed no salient differences. As presented in Fig. 1B, N17, N78, N98, N247, and N299 were highly conserved in all strains. Other sites were not observed in all isolates.

**FIG 2:**
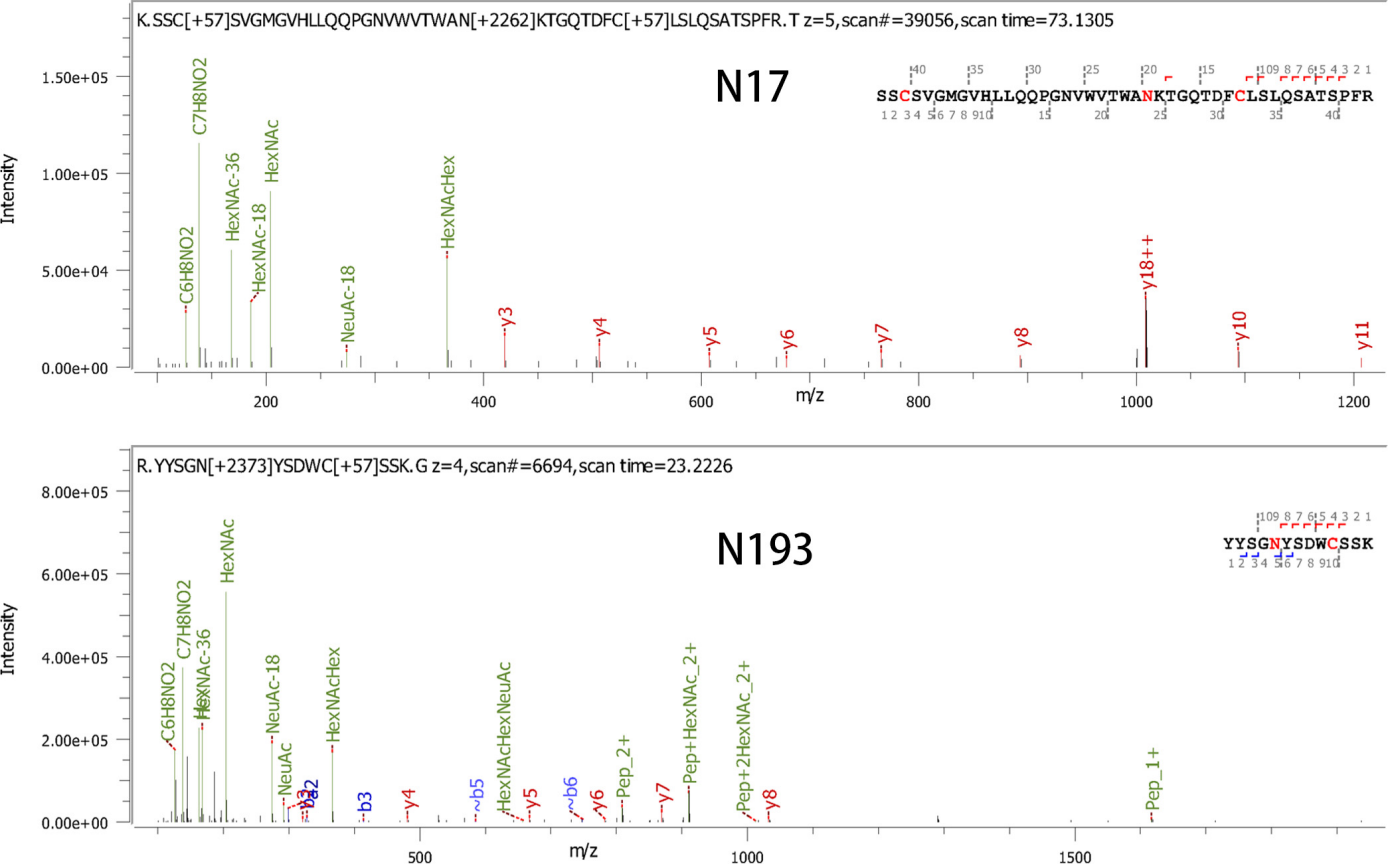
Identification of NGSs in gp85 protein of ALV-J strain TBC-J6 through LC-MS. Representative LC-MS data of two glycosylation peptides (containing N17 or N193) in gp85-rIgG. The b-ions and y-ions in the spectrum are shown.

In addition, to investigate the existence of NGSs in gp85 of TBC-J6, we purified the protein gp85 fused to rabbit IgG-Fc and applied liquid chromatography-mass spectrometry (LC-MS) in our study. Fig. 2 showed the representative LC-MS data of two glycosylation peptides (containing N17 or N193) in gp85-rIgG. All the peptides containing N-glycosylation were identified (see Table 1). Those results proved our hypothesis that the glycan chains exist in each predicted NGS.

**TABLE 1:** Summary of identified NGSs and glycan chains on ALV-J gp85 protein through LC-MS

N-linked glycosylation sites	Identified peptides	Peptide no.	Type of N-glycan chains			Avg mass of N-glycan chain
			High-mannose form	Hybrid form	Complex form	
N17	*KSSCSVGM*^1^GVHLLQQPGNVWVTWANKT^19^	67	0.00% (2)	16.67% (12)	83.33% (53)	2906.12
N98	^97^KN*GTKRTCVTFGSVCYKE^114^	299	3.68% (11)	6.02% (18)	90.30% (270)	2276.83
N116	^102^RTCVTFGSVCYKENN*RS^118^	222	2.25% (5)	9.91% (22)	87.84% (195)	2333.51
N131	^121^RVCHIFDGNFN*GTGGAEAELRD^142^	332	4.22% (14)	14.46% (48)	81.33% (270)	1915.74
N157	^146^KWKGNDHLIRPYVN*QSWTMVSPINTESFSISSRY^179^	451	9.31% (42)	13.75% (62)	76.94% (347)	2898.52
N185	^178^RYCGFTSN*ETRY^189^	95	7.37% (7)	17.89% (17)	74.74% (71)	1664.23
N193	^188^RYYSGN*YSDWCSSKG^202^	159	3.77% (6)	15.09% (24)	81.13% (129)	1958.03
N209	^201^KGGEWSGGN*CTAEWNYYAYGFTFRT^225^	134	4.48% (6)	14.93% (20)	80.60% (108)	1867.90
N226	^224^RTN*ESEVLWNNGTAKA^239^	12	33.33% (4)	25.00% (3)	41.67% (5)	2478.41
N234	^224^RTNESEVLWNN*GTAKA^239^	2	0.00% (0)	0.00% (0)	100.00% (2)	1939.18
N274	^258^RNALGGPCYLGQLTMLSPN*FTTWMTYGPNITGHRR^292^	47	25.53% (12)	72.34% (34)	2.12% (1)	1438.75
N284	^258^RNALGGPCYLGQLTMLSPNFTTWMTYGPN*ITGHRR^292^	68	5.88% (4)	22.06% (15)	72.06% (49)	1814.13
Total		1888	5.99% (113)	14.56% (275)	79.45% (1500)	2267.54

The italicized portion of the sequence represents signal peptide ahead gp85.

N* represents identified N-glycosylation sites from LC-MS.

Superscript numbers represent positions of amino acids in gp85-rIgG.

### Generation of ALV-J strains with mutated NGSs.

**FIG 3:**
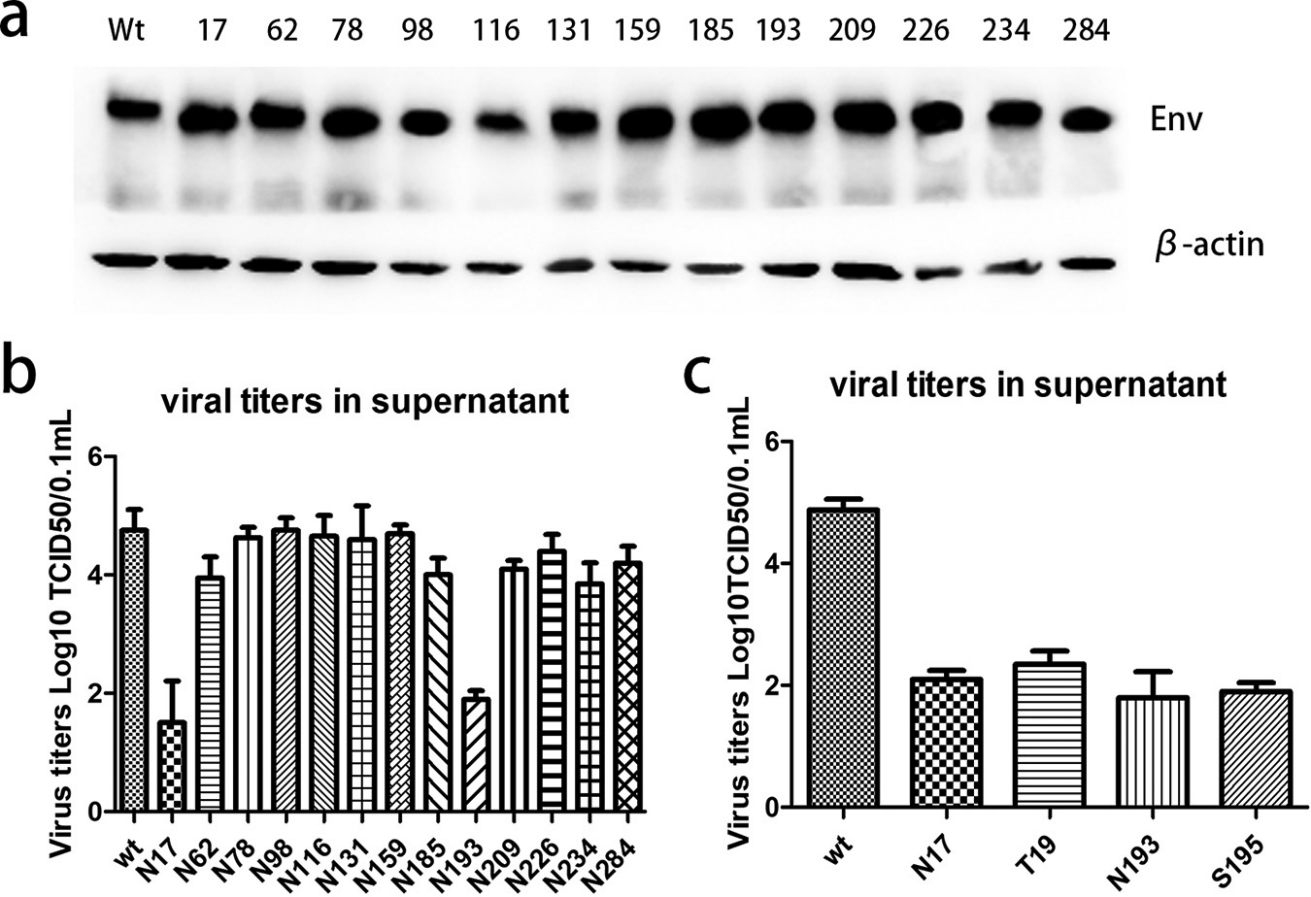
Generation of ALV-J strains with mutated NGSs. (a) Western blot analysis of wide-type and13 mutant ALV-J strains. β-actin was included as a loading control. (b) Viral titers of wide-type and 13 mutant ALV-J strains after 3 continuous passages in DF-1 cells. (c) Viral titers of wide-type, N17, T19, N193, and S195 ALV-J strains after 3 continuous passages in DF-1 cells.

We mutated asparagine to tryptophan at 13 sites in the *gp85* of TBC-J6 to ensure that the smallest change was introduced on the molecular surface because both asparagine and tryptophan were polar amino acids. Four days after transfection with various infectious clones, we harvested the culture supernatant containing the virus stocks and inoculated them into secondary DF-1 cells. After three passages in DF-1 cells, they were positive for both Env and p27 using immunofluorescence (IFA). These results indicated that all the mutations in the predicted NGSs were tolerant for ALV-J. Fig. 3A shows the expression of Env protein of different mutant strains where different decreases in the Env protein size were observed in different mutant strains. All mutants had salient lower bands compared with the wild-type virus. This could result from the loss of glycan chains.

The viral growth of each mutant was tested after three passages of the infected DF-1 cells. It was found that most of the mutant viruses had no significant difference in the virus titer within DF-1 cells except for two strains, N17, and N193 mutants, which had viral titers reduced by more than 500-fold compared to that of the wild-type virus (Fig. 3B). To further confirm that the changes were the result of nonglycan chains rather than the substituted amino acids, we also mutated the T19 and S195. The results substantiated the presumption that the mutants N17Y and T19A, N193Y, and S195A had similar viral titers (Fig. 3C).

### Single N193 glycosylation is responsible for high-level viral growth.

**FIG 4:**
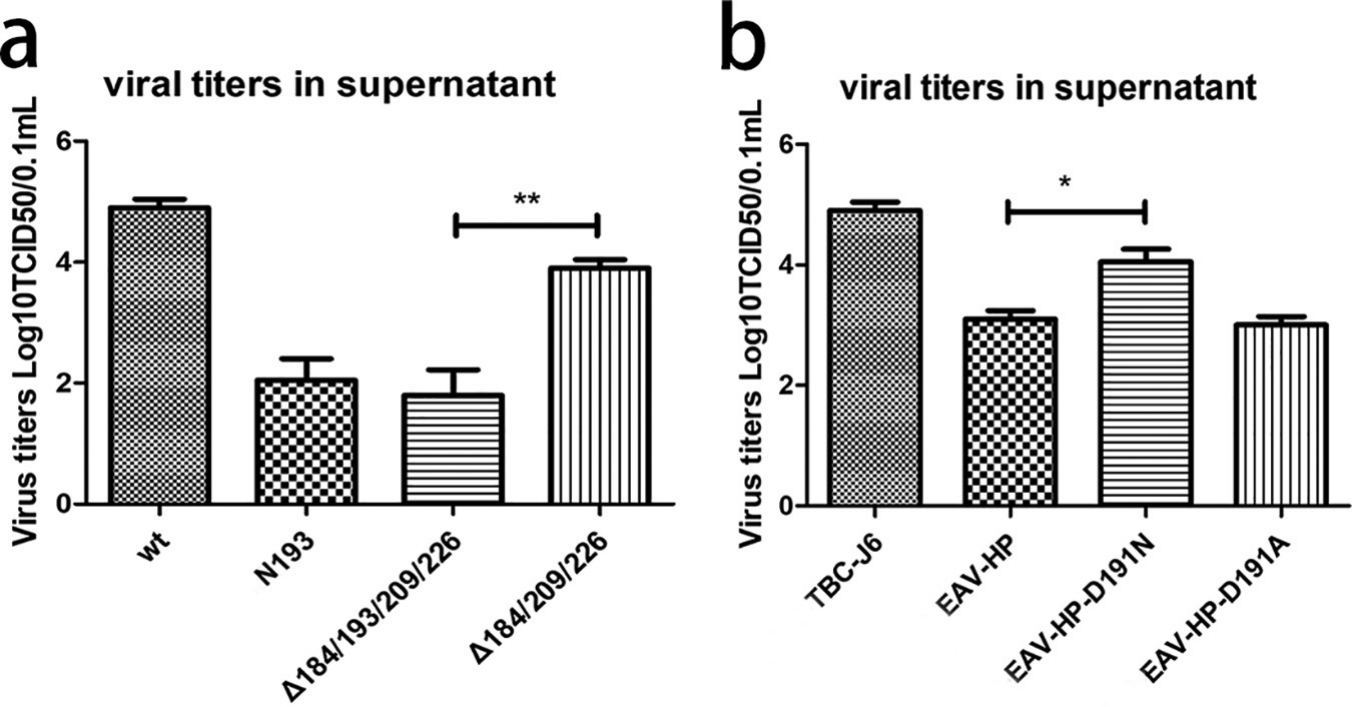
Phenotype of ALV-J strain TBC-J6 with multiple mutations in the NGSs and EAV-HP. (a) Viral titers of wide-type, N193, Δ185/193/209/226, and Δ185/209/226 after 3 continuous passages in DF-1 cells. (b) Viral titers of TBC-J6, EAV-HP, EAV-HP-D191N, and EAV-HP-D191A after 3 passages in DF-1 cells.

In the hr2 region of *gp85* of TBC-J6, there was 2 NGSs present at positions 193 and 209. Of those, only the mutation in N193 could result in a reduced virus titer. To elucidate whether adjacent NGSs also contributed to the observed reduction of virus titer that was induced by N193 mutation, we constructed two more infectious clones with multiple mutations, Δ185/193/209/226 and Δ185/209/226, and with the same mutation mentioned above at each corresponding NGS. Consistent with previous results, Δ185/193/209/226 had almost the same titer as N193 mutant while Δ185/209/226 was similar to the N185 mutant, indicating that N193 was the pivotal NGS that could solely affect the fitness of the ALV-J strain TBC-J6 and that the glycosylation status at N185, N209, and N226 was dispensable (Fig. 4A).

To study whether N193 could promote the proliferation of viruses that originally lacked this residue, we replaced the *gp85* of EAV-HP with the corresponding region of TBC-J6. A viral titer assay indicated that EAV-HP-D191N had a 5-fold higher titer than EAV-HP and EAV-HP-D191A (Fig. 4B).

### N193 glycosylation was crucial for virus-receptor binding.

**FIG 5:**
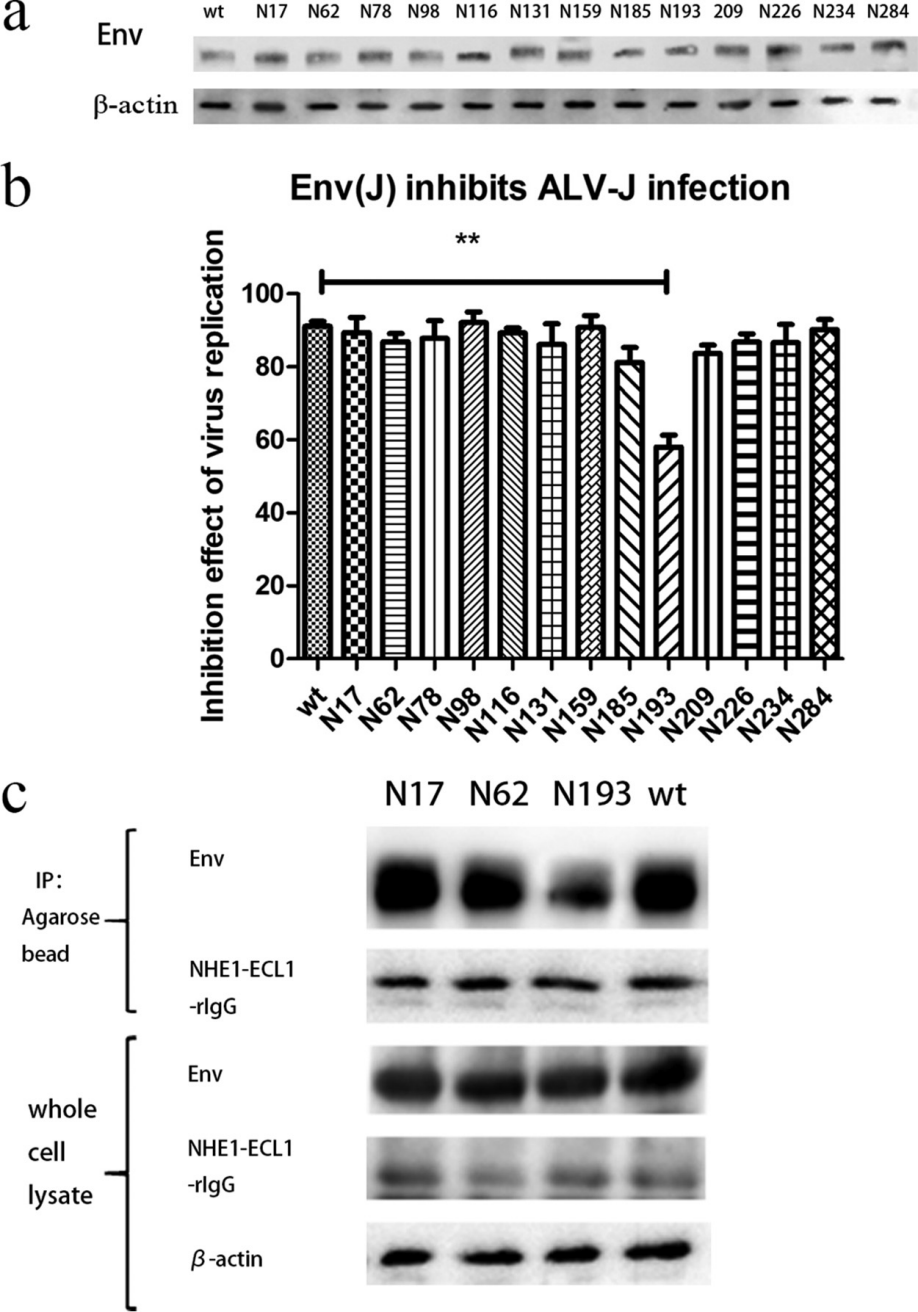
Analysis of Env-receptor binding affinity through blocking assay of viral entrance and coimmunoprecipitation. (a) Expression of various mutant Env proteins and β-actin in the lysates of DF-1 cells transfected with different plasmids. (b) The blocking ability of various pCAGGS-Env vectors on ALV-J replication and the wide-type was regarded as the standard. (c) Detection of the affinity of receptor NHE1-ECL1-rIgG binding with various mutant Env proteins through coimmunoprecipitation. Env protein and β-actin in the cell lysates were regarded as references.

To explore whether a mutation at N193 affected interactions between Env and NHE1, we first optimized the transfection condition of Env expression plasmid in DF-1 cells. When DF-1 cells were transfected with 250 ng of pCAGGS-env(wt) for 2 days, there was a 91% reduction of ALV-J infection, which was assessed by measuring the mRNA level. We used 250 ng for subsequent transfections with various mutant pCAGGS-Env plasmids to ensure that all the mutant Env proteins were expressed at the same level. Half of the cells of each sample were subjected to Western blot. The result clearly showed that a similar amount of Env was expressed in all the transfections (Fig. 5A). The inhibition effect of various mutant Envs on virus infection was tested by the same method. As shown in Fig. 5B, the majority of the Env protein expression vectors could eliminate ALV-J expression to a large extent ranging from 85.2% to 96.5%. However, the N193 mutation could only achieve an inhibition rate of less than 60%.

In addition, the coimmunoprecipitation assay was carried out to detect interactions among receptor and Env proteins. NHE1-ECL1-rIgG, the first extracellular loop of the ALV-J receptor NHE1 that was fused to rabbit IgG Fc, was predicted to bind to all the mutant Env proteins. We found that NHE1-ECL1-rIgG could only pull down a small amount of N193-mutant Env protein in the lysate of transfected DF-1 cells in contrast to other counterparts (Fig. 5C). A gray value analysis indicated a significant difference between the N193-mutant Env protein and others. The results indicated a lower binding affinity between N193-mutant Env protein and ALV-J receptor NHE1.

**FIG 6:**
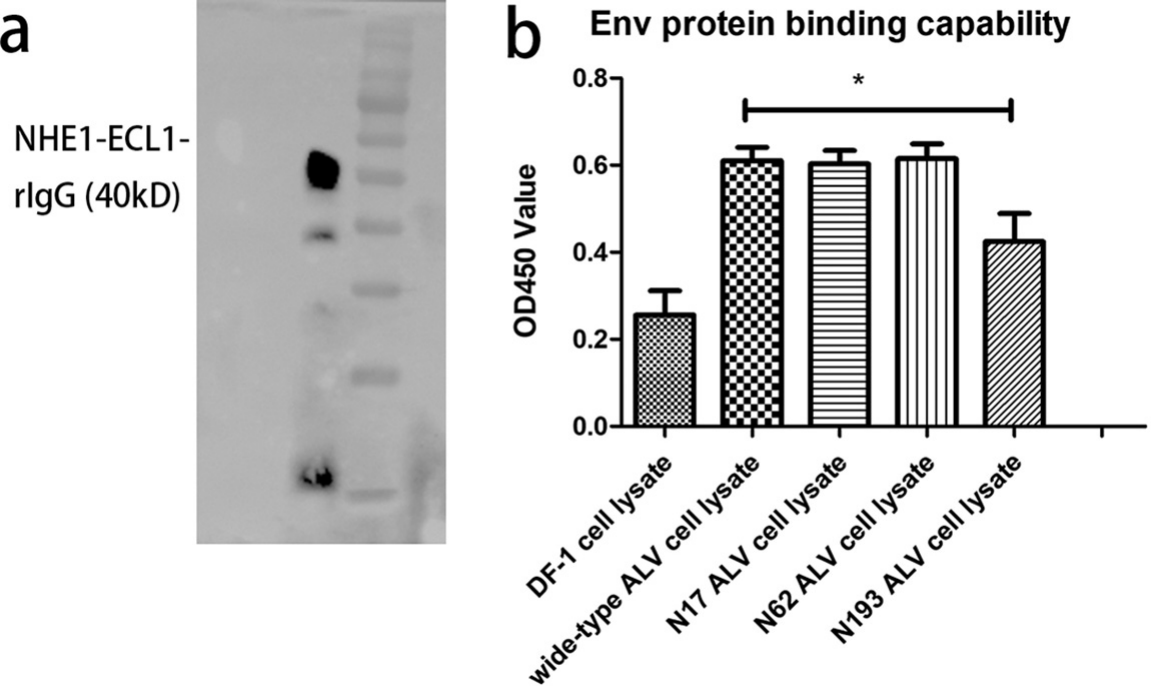
Analysis of Env-receptor affinity through ELISA. (a) Western blot analysis of the soluble chNHE1-ECL1-rIgG. (b) Detection of affinity of various mutant Env expression cell lysates with NHE1-ECL1-rIgG using ELISA. The binding capability was measured by OD_450_ values of negative DF-1 cells, wide-type, N17, N62, and N193.

To further confirm the difference in binding affinity, we developed an enzyme-linked immunosorbent assay (ELISA) method to evaluate the interaction of Env-NHE1. Fig. 6A shows that the purified NHE1-ECL1-rIgG was detected by Western blot. DF-1 cell lysates from Env expression plasmid transfection were added to each well coated with the NHE1-ECL1-rIgG. Anti-gp85 MAb JE9 was later used to capture the Env protein. Fig. 6B shows that the optical density at 450 nm (OD_450_) was nearly the same in the wide-type, N17, and N62, whereas it was only slightly higher for N193 compared to uninfected DF-1 cells. These results indicated that the N193-mutant Env protein interferes with the binding to the NHE1 receptor of ALV-J.

### N17 affected ALV-J by influencing Env protein processing and virion incorporation.

**FIG 7:**
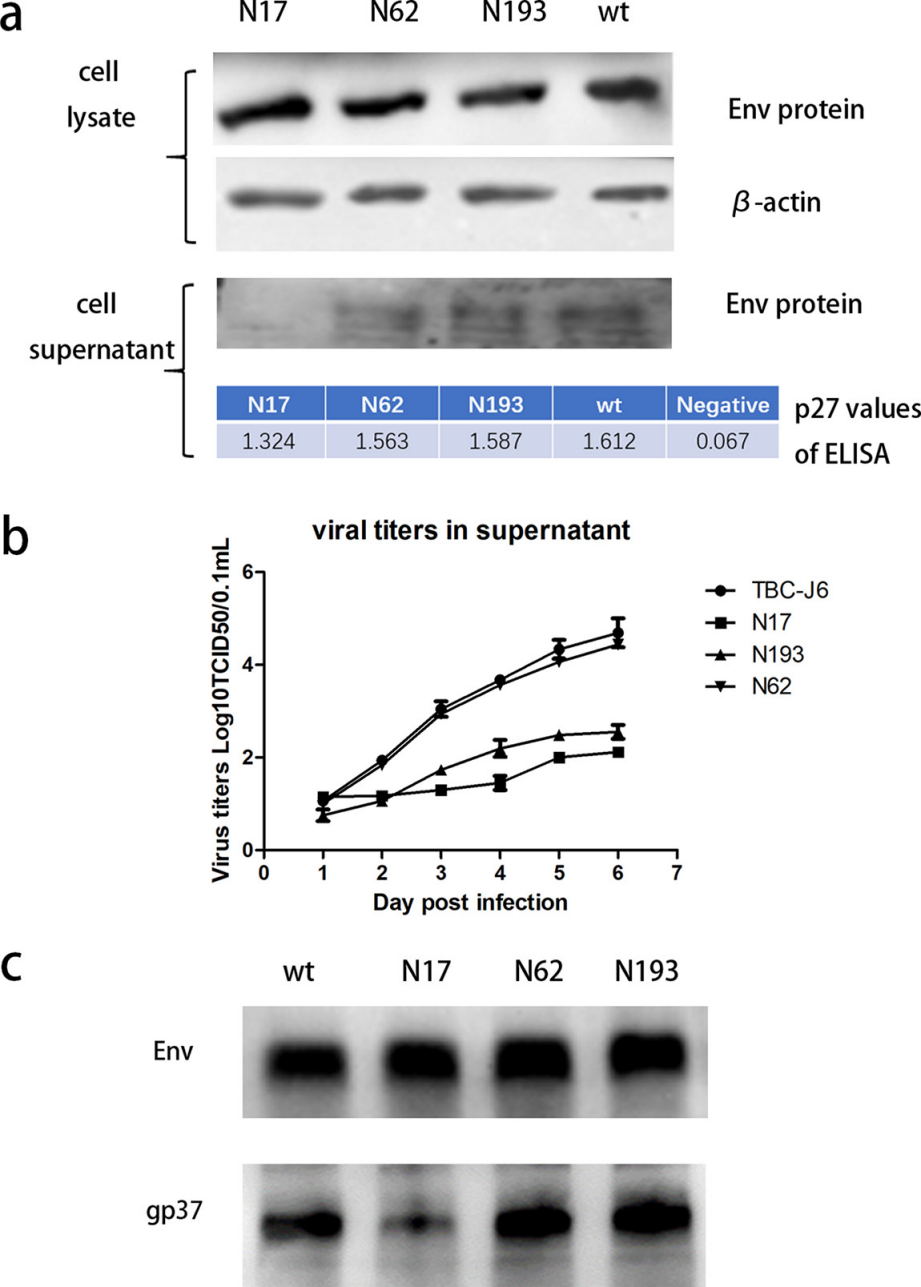
Detection of the ability of virion incorporation and proteolytic process of various mutant ALV-J strains. (a) Detection of virion incorporation through quantifying Env protein and p27 in the supernatants of wide-type, N17, N62, and N193 mutants. Env protein and β-actin in the cell lysates were regarded as references. Env protein and β-actin were detected by Western blot while p27 was detected by ELISA. (b) Supernatants of wide-type, N17, N62, and N193 mutants were harvested at 1 to 6 days postinfection and then tittered for TCID_50_ using the Spearman-Kärber method. (c) Env protein processing was quantified by detecting *gp37* in the cell lysates of wide-type, N17, N62, and N193 using JE9 and rabbit anti-*gp37* antibodies.

To explore whether the decreased virus titer was caused by the effect of the NGS in Env protein incorporation into virions, Env and p27 in the supernatant were quantified. To ensure that p27 and Env protein in the supernatant was not affected by the number of infected cells and Env-receptor affinity, we retained the infected cells for 7 days to ensure all cells were infected. N62 served as a negative-control along with the wide-type strain. By analyzing the Env protein in the infected cell cultures, we found that the 4 samples had almost the same amount of Env protein expression in the infected cell lysate (Fig. 7A, upper panel). In the supernatant, the N62 and N193 mutants had almost the same level of p27 (ELISA) and Env protein (Western blot) as the wild-type virus. However, the N17 mutant had less Env protein, although it was with a slightly smaller amount of p27 (Fig. 7A, lower panel). This indicated that N17 might affect Env protein incorporation into viruses. To test this hypothesis, the proliferation rates of 4 strains were examined by measuring the virus titer during the 6 day infection period. Consistent with previous results, N17 and N193 mutants had a lower virus replication rate than wild type and N62 mutant (Fig. 7B). To assess whether the inability of N17 to aid incorporation into virions resulted from the proteolytic process of Env precursor protein, we examined the expression level of *gp37* in each sample. Results in Fig. 7C show that the 4 ALV-J strains had the same amount of Env protein in the cell lysates whereas the N17 mutant yielded less *gp37* than the other 3 strains. A gray value analysis showed the gp37 protein in the N17-mutant ALV-J infected cells was significantly lower than that in other samples. In summary, our results indicated that a mutation in N17 decreased the efficiency of Env protein processing.

## DISCUSSION

The envelope protein is essential in the life cycle of ALV in that it directly interacts with the host receptor and promotes the invading process. Previous studies indicated that *env* gene sequences of ALV-J isolates are related to the genetic background of their host chickens (8, 23). NGSs in Env proteins of different strains are also linked to their background. Thus far, most molecular epidemiology studies have focused on the primary structure of Env. Although glycosylation is important for ALV-J fitness, little research had been done in this field. In this report, we studied the function of NGSs in ALV-J in multiple ways.

In contrast to the highly stable NGSs in ALV of subgroups A-E (24), it was found that many NGSs are not presented in all the ALV-J strains. It indicated that these nonconserved NGSs may not be essential for ALV-J. In this study, TBC-J6 was chosen for further analysis because it has a large-scale deletion in hr2 as well as different NGSs that were thought to be responsible for receptor-binding. In addition, it belongs to the phylogenetic tree branch that is prevalent in China. TBC-J6 replicated faster and had competitive advantages over other ALV-J strains (22).

To determine the role of NGSs in Env function, we mutated each of the NGS in *gp85* individually. All 13 mutant viruses kept the ability to infect DF-1 cells, while two strains, N17 and N193 mutants, largely decreased their viral titer. In general, glycosylation plays a crucial role in protein folding, structure, sorting, trafficking, and stability (25). We found that 100% of ALV-J strains had N17, while only 93.8% had N193 (N191, N190). Importantly, the prototype strain of ALV-J, HPRS103, lacks N193. We asked whether gaining or losing N193 affected the fitness of HPRS103 or whether some strains lack it. To study whether N193 was a common factor that contributed to the ALV-J titer, we used EAV-HP, the endogenous virus that is thought to be the ancestor of ALV-J (26). The Env sequence of EAV-HP shared 98.5% identity with HPRS103 and EAV-HP also lacked N193 (N191). When adding one NGS at N191 (N193) of EAV-HP Env, the virus titer increased by 5-fold. This means that glycan chains in this region do affect different ALV-J strains. In addition, it has been reported that, when multiple glycosylation sites are close, there exists competition of glycan addition. If mutation at one site occurs, the adjacent ones can compensate for its function (27). To test this theory, a series of multiple-NGS mutant ALV-J strains were constructed. Results of our subsequent experiment contradicted this theory because only the N193 mutation largely affected the ALV-J replication. In conclusion, glycosylation at N193 is a common factor that can solely affect the titer of ALV-J.

Theoretically, when Env proteins bind to the receptors on the cell surface, they can block the ALV-J infection process. In the blocking assay of the viral entrance, the N193-mutant Env protein caused a lower level of blockage, indicating its lower affinity with the ALV-J receptor. Thus, we suspected that N193 may be crucial in receptor binding. To test this hypothesis, we used coimmunoprecipitation (co-IP) to examine the mutant Env proteins’ capacity to bind to the receptor NHE1. Reduced N193-mutant Env protein was pulled down when compared with others, suggesting that the NGS at position 193 affects the receptor binding affinity. Later, we carried out solid-phase binding ELISA, as reported previously (18), to detect the affinity of various mutant Env proteins with the receptor NHE1 based on anti-*gp85* MAb JE9. JE9 targets at the 83^rd^ to 88^th^ aa of *gp85* but is not a neutralizing antibody (28). This means it binds to a none-receptor-binding site, and this ELISA method was feasible for our purpose. Results indicated that reduced N193-mutant protein was captured by this system, which confirmed our hypothesis that N193 is indispensable for Env to bind to NHE1.

The amino acids 38 to 131 and 159 to 283 of *gp85* in ALV-J are identified as the receptor binding sites (18). However, mutations in N17 and S19 did reduce the viral titer to a large extent. Consequently, we suspected that another mechanism may cause this phenomenon. A previous study demonstrated that the single mutation at the 2^nd^ or 6^th^ NGS of the ALV-A Env protein could dramatically impair the virus fitness by influencing the folding process and virion incorporation of Env protein (24). In addition, we found that although Env protein in ALV-J shows only 40% homology in amino acid compared with those from other subgroups (ALV-A-E), the first 5 NGSs of ALV-J Env protein, which lies at position 17, 56 (62), 78, 98 and 116, are highly positional conserved with the counterparts at 17, 59, 80, 97, 117 from ALV-A-E. Taken together, it is possible that a mutation at N17 can also influence virion incorporation into ALV-J.

Here, we referred to the studies using HIV-1 pseudoviruses (29, 30) where the viral pellets were subjected to p24 and gp120 ELISA, and the viral incorporation was determined by calculating the ratio of gp120 to p24 (31). By analyzing the Env protein and p27 in the cell culture supernatants, we found that the N17-mutated virus had a slightly smaller amount of p27 but much less Env protein than other samples. Because N17 is not involved in receptor binding, the lower viral replication could be explained by the defect in virion incorporation. Later, the Env protein processing had also been tested. A smaller amount of the *gp37* suggested that N17 mutation could affect the proteolytic process of Env protein. After being glycosylated and folding, Env proteins enter the Golgi apparatus for cleavage into *gp85* and *gp37* and are finally transported to the cell surface. However, mutation at the highly conserved N17 greatly affected the efficient proteolytic process, further reducing virion particles released into the supernatants. Two explanations are plausible. It has been reported that many glycoproteins are not tolerant to the removal of glycosylation sites close to N termini because these sites are first engaged in the ER protein folding machinery to promote the proper folding process (32, 33). Because N17 is the first NGS in *gp85*, this theory is reasonable. Disulfide bonds are another important factor. Mass spectrometry indicated that in Env (ALV-A), a disulfide bond links the two subunits through the first cysteine residue Cys25 in *gp85* and the last extracellular cysteine Cys438 in *gp37* (34). Those two cysteines are conserved in all ALV subgroups, including ALV-J. Because Cys25 is close to N17, it is possible that the glycan chains at N17 can affect this disulfide bond. When N17 is mutated, the Env protein may alter its second structure, thus inhibiting the proteolytic process.

The importance of NGSs in ALV-J is far beyond what we concluded in this study. Unlike HPRS103, many ALV-J strains lack the 6th (N116) and 11th (N209) NGSs (18), such as CAUHM01, JS09GY3, HAY013, and GD13GZ. Although we identified N193 as being crucial, only 93.8% of ALV-J strains had it. We believe the small portion of other ALV-J strains had similar results, like HPRS103 relying on N209 because N209 and N193 are close to each other. In essence, this phenomenon is caused by the shift of receptor binding domains between NHE1 and *gp85*. *Gp85* has shown extensive variability among different ALV-J strains. The host range of ALV-J has gradually expanded, together with the more and more complex pathogenicity of the virus reported in China, all of those are tightly linked to *gp85*. Providing the fast-evolving *gp85*, studying its primary structure and NGSs is especially important.

Our study systematically analyzed the relative importance of each NGS in the *gp85* of ALV-J. It was found that not all the glycan chains are essential for ALV-J fitness. N17 was responsible for Env protein processing and virion incorporation while N193 plays a critical role in receptor binding. These findings should be valuable for our understanding of the roles of the glycans in ALV-J infection as well as providing potential anti-ALV-J strategies.

## MATERIALS AND METHODS

### Viruses and cells.

293T and DF-1 cells were maintained in Dulbecco’s modified Eagle’s medium (DMEM; Thermo Scientific, USA) supplemented with 10% fetal bovine serum (FBS; Gibco, USA). ALV-J strain TBC-J6 was isolated from Tibetan chicken (22). ALV-J clone containing the *gp85* of EAV-HP was generated in our laboratory (35).

### Prediction of NGSs in different ALV strains and data analysis.

*Env* gene sequences of ALV-J strains with different backgrounds were obtained from GenBank. Prediction of the potential NGSs was carried out online (http://www.cbs.dtu.dk/services/) as described previously (36, 37). A score of 0.5 was set as a threshold. It was regarded as positive when the score was above 0.5. Statistical analysis was performed using GraphPad Prism 5 software (GraphPad Software, Inc.). *P* < 0.05 was considered statistically significant. ImageJ (National Institutes of Health) was used to analyze the gray value of Western blot results (38).

### Construction of infectious clones of ALV-J and protein expression vectors.

The infectious clones of ALV-J with different mutated NGSs were constructed using a site-directed mutagenesis approach. For this, 13 pairs of primers (Table S2) were designed for those predicted sites. PCR was carried out to amplify the full-length plasmid of the infectious clone of ALV-J pMD-TBC-J6 with the 13 pairs of primers, respectively. The linearized pMD-TBC-J6 was recombined with the commercial recombinant enzyme ExnaseTM II (Vazyme, Jiangsu, China) as instructed. Recombinant plasmids were then transfected into DF-1 cells with 70% confluence using TransIT-LT1 transfection reagent (Mirus, Madison, WI) according to the supplier’s instructions. Four days later, the supernatant was passed into fresh DF-1 cells and incubated for 5 days. After three passages in DF-1 cells, indirect immunofluorescence (IFA) was carried out to detect the expression of *gp85*. The introduced mutations for both constructed plasmids and the cDNA of infected DF-1 cells were confirmed by Sanger sequencing.

To construct Env protein expression vectors, one pair of primers was designed to amplify the whole Env gene. Thirteen mutant infectious clones of pMD-TBC-J6 described above were used as the templates. The PCR products and the pCAGGS vector were digested by EcoRI and XhoI (Thermo Fisher Scientific, Waltham, MA). The *Env* genes were subsequently inserted into the pCAGGS backbone. The plasmids were transfected into DF-1 cells and then tested by IFA 2 days after transfection.

### Western blot and coimmunoprecipitation.

Proteins were separated on 12.5% SDS-PAGE gels and transferred onto a nitrocellulose membrane. After blocking in 5% skimmed milk at 37°C for 1 h, the membrane was incubated with anti-*gp85* MAb JE9, anti-β-actin MAb (Abcam, Cambridge, UK) or rabbit anti-*gp37* antibody (39) for 1 h at 37°C. After being washed three times with phosphate-buffered saline containing 0.05% Tween 20 (PBST), the membrane was incubated with HRP-labeled anti-mouse IgG (H+L) antibodies or anti-rabbit IgG (H+L) antibodies (Jackson, PA, USA), for 1 h at 37°C. Finally, the protein bands were developed with NcmECL Reagent (NCM Biotech) and scanned using a FluorChemE imaging system (Protein Simple, CA, USA).

To detect the binding affinity of various mutant Env proteins with ALV-J receptor NHE1, DF-1 cells cultured in 6-well plates were transfected with the various plasmids expressing mutant Env and the pCAGGS-NHE1-ECL1-rIgG, which expresses the first extracellular loop of ALV-J receptor NHE1 fused to rabbit IgG Fc. Two days after transfection, cells were washed three times with PBS and then lysed with 200 μL of the Co-IP lysates (50 mM Tris, 150 mM NaCl, 1% NP-40, 0.25% Sodium deoxycholate) containing PMSF (0.1 mM) at 4°C. After centrifugation, 30 μL of the lysates was removed as the input fraction. Thirty microliters of the protein A/G Agarose beads (Santa Cruz Biotechnology, TX) were added into the remaining supernatant and incubated at 4°C overnight. The beads were washed five times with PBS and boiled with SDS-loading buffer for 10 min before analyzing by Western blot with the indicated antibodies.

### Viral entrance blocking assay.

The blocking assay was performed to evaluate the blocking effect of various Env proteins on ALV-J entry. First, we transfected DF-1 cells in a 12-well plate with different amounts of plasmids encoding the wide-type Env described above. Two days after transfection, cells were inoculated with ALV-J stock (1000 TCID_50_). Three days later, we examined the relative expression of ALV-J using real-time quantitative PCR (qPCR) on the *p27* gene. The cycling conditions were 95°C for 30 s followed by 40 cycles of 95°C for 5 s and 59°C for 34 s.

Based on the optimization result, 250 ng of plasmids was used for transfection subsequently. DF-1 cells in a 12-well plate were transfected with 250 ng of various mutant Env protein expression vectors. Later, cells were treated as described above. Each experiment was performed independently three times.

### Detection of *gp85* incorporation into virions.

First, we inoculated the DF-1 cells in the 12-well plates with various mutant ALV-J strains (1000 50% tissue culture dose [TCID_50_]) and incubated them for 7 days to ensure high levels of infection, which was confirmed by IFA. The supernatant and cell lysate of each infection were then collected separately. Using Western blot analysis, we detected the expression level of Env protein in the cell lysates. Afterward, Env protein and p27 in the supernatant were detected by Western blot and ELISA, respectively. The specific procedure of sandwich ELISA was taken from a previous publication (40).

### Viral growth kinetics detection.

All culture supernatant samples were titrated for TCID_50_ using the Spearman-Kärber method. To detect the viral growth kinetics, DF-1 cells were inoculated with various mutant ALV strains at a multiplicity of infection (MOI) of 0.1 in each well. One well of each sample was harvested and titrated daily at 1 to 6 days postinfection as described above.

### Detection of Env-NHE1 binding affinity through ELISA.

We transfected 293T cells with pCAGGS-NHE1-ECL1-rIgG and harvested the supernatant 2 days after transfection. After being filtered by a 0.22 μm filter, the fusion protein NHE1-ECL1-rIgG was harvested and concentrated through HiTrap^TM^ Protein G HP (GE Healthcare, Uppsala, Sweden). The purified protein was confirmed by SDS-PAGE and Western blot using HRP-labeled anti-rabbit IgG antibodies. Then, 96-well ELISA plates were coated with 0.5 μg/mL fusion protein NHE1-ECL1-rIgG for 16 h at 4°C and blocked with PBST containing 5% skimmed milk for 4 h. Afterward, plates were washed with PBST twice. Samples to be tested are the lysates of DF-1 cells infected with various ALV-J strains for 7 days and harvested by the freeze-thaw method.

To detect the binding ability of different mutant Env proteins with the receptor NHE1, different samples were added to the plates mentioned above for 1 h at 37°C. After incubation with cell lysates, the wells were washed 3 times with PBST and incubated with a 1:2000 dilution of the JE9 for 1 h at 37°C. The wells were washed 3 times with PBST and incubated with a 1:30000 dilution of the HRP-labeled goat anti-mouse IgG antibodies for 1 h at 37°C. Finally, after 3 to 5 washes with PBST, 100 μL of substrate solution was added to develop color for 15 min, and the reaction was stopped by adding 50 μL of 2 M H_2_SO_4_ to each well. The absorbance at 450 nm was measured using an ELISA reader.

### Detection of NGSs in TBC-J6 gp85 protein through LC-MS.

The TBC-J6 gp85 sequence fused to rabbit IgG-Fc was inserted into the pCAGGS vector. We transfected DF-1 cells with pCAGGS-gp85-rIgG and harvested the fusion protein 2 days after transfection just the same way as purification of NHE1-ECL1-rIgG mentioned above. LC-MS analysis was performed using Thermo Scientific EASY-nLC 1000 System (Nano HPLC) (Thermo Scientific) connected to Orbitrap Fusion Lumos Tribrid (Thermo Fisher Scientific, Bremen, Germany). Filter Aided Sample Preparation (FASP) was fulfilled by collecting concentrated peptides and trypsin digestion. Peptides containing glycan chains were enriched by the HILLIC column. Then the samples were subjected to the Thermo Scientific EASY-nLC 1000 System. The acquired MS raw data were identified and quantitatively analyzed by byonic software (Protein Metrics Inc.).
