# Supplementary material for: Glycosylation of ALV-J Envelope Protein at Sites 17 and 193 Is Pivotal in the Virus Infection
Source: J Virol. 2022 Feb 23;96(4):e01549-21. doi: 10.1128/jvi.01549-21 (PMC8865534; doi:10.1128/jvi.01549-21)
Supplement: Supplemental file 1 — Tables S1 and S2. Download jvi.01549-21-s0001.pdf, PDF file, 0.03 MB [file jvi.01549-21-s0001.pdf]

Table S1 ALV-J strains used for analyzing NGSs

Isolate	Accession no.	Isolate	Accession no.	Isolate	Accession no.
**HPRS103**	Z46390	**SDAU1102**	KU159178	**GX20YL05**	MW491266
**NX0101**	AY897227	**SD1109**	KF768000	**HN1001-2**	HQ260975
**Js-nt**	HM235667	**WB1106j**	JX570795	**GX12NN20**	KT598483
**JS09GY3**	GU982308	**WB11058J**	JX570793	**NHH**	HM235668
**SCAU-HN06**	KQ900844	**WB11098(J)**	JX848322	**PK19SA01**	MN956380
**TBC-J6**	MT409625	**SD100502J**	HQ270188	**10022-16**	GU222400
**TBC-J4**	MT409624	**HLJ09SH02**	HQ634807	**LN08SY10**	HQ634802
**GD1407**	KU500034	**SDAU1704**	KY980660	**SCAU11-XG**	KC149971
**GX14HG01**	KU997685	**DBYJ1102**	KC875858	**GD1109**	JX254901
**GD18QY22**	MN262620	**GX20YL09**	MW491270	**JL1801**	MN419348
**GD14J2**	KU500032	**WSC112**	KJ631322	**YZ9902**	HM235670
**GD17FS01**	MN262599	**HAY013**	HM235665	**Hc1**	AF247391
**JX19ND03**	MN262539	**NM2002-1**	HM235669	**SDAU1702**	KY980658
**JX19TH01**	MN262528	**IMC10200**	AY234051	**FJ201306**	KM655820
**JL1802**	MN419349	**SDC2000**	AY234052	**SCMZ92**	KF796647
**GD13GZ**	KU500030	**GD17ZQ06**	MN262604	**HuB09JY04**	JN378888
**WGZ13**	KJ631313	**SD9901**	AY897220	**ZB110604-6**	KC841157
**JL1803**	MN419350	**4817**	AF247385	**ZB110604-5**	KC841156
**XX2-09**	HM775331	**SD0002**	AY897224	**ZB110604-3**	KC841154
**SD1804**	MN419338	**SD9902**	AY897221	**GX14HG03**	KY983569
**GM209-4**	MH379645	**JS1807**	MN419341	**CAHHM01**	HM640944
**GM209-5**	MH379646	**SDMG1801**	MN413674	**JS09GY6**	GU982310
**HB1801**	MN419352	**TJ1801**	MN419353	**HLJ13SH01**	KM376510
**LH20180301**	MK944404	**GD18JM01**	MT538241	**HB2010001**	MZ197985
**SD1810**	MN419347	**JS14NT02**	MN735302	**JL093-1**	JN624878
**JS1808**	MN419342	**CAUSY01**	JF932001	**JL08CH3-1**	HQ634809
**JS1806**	MN419340	**sdau1002**	JN389518		

Table S2 Primers used for constructing mutant infectious clones of ALV-J

Primer	Sequence
**N17**	TGGGTCACCTGGGCATATAAGACGGGCCAAAC
	GTTTGGCCCGTCTTATATGCCCAGGTGACCCA
**N62**	GCTACTGCTTGCGAATATGACACCGATTTAGC
	GCTAAATCGGTGTCATATTCGCAAGCAGTAGC
**N78**	ATGTTTAATACAGACTCTATATACGACCCTCCCTTG
	CAAGGGAGGGTCGTATATAGAGTCTGTATTAAACAT
**N98**	GGTCCCAAATGATCAAGTATGGAACAAAACGTACGTG
	CACGTACGTTTTGTTCCATACTTGATCATTTGGGACC
**N116**	TGTGCTATAAAGAGAACTATCGCAGTAGCAGTAG
	CTACTGCTACTGCGATAGTTCTCTTTATAGCACA
**N131**	CATTTTTGATGGGAATTTTTATGGGACTGGTGGGG
	CCCCACCAGTCCCATAAAAATTCCCATCAAAAATG
**N159**	CTTATAAGACCCTATGTCTATCAATCATGGACGATGGT
	ACCATCGTCCATGATTGATAGACATAGGGTCTTATAAG
**N185**	TTGTGGATTCACCAGCTATGAGACTCGTTATTATAGC
	GCTATAATAACGAGTCTCATAGCTGGTGAATCCACAA
**N193**	CTCGTTATTATAGCGGGTATTATTCCGATTGGTGTAG
	CTACACCAATCGGAATAATACCCGCTATAATAACGAG
**N209**	GAATGGTCAGGTGGTTATTGCACGGCGGAAT
	ATTCCGCCGTGCAATAACCACCTGACCATTC
**N226**	GTTTACCTTCAGGACGTATGAGTCAGAGGTGTT
	AACACCTCTGACTCATACGTCCTGAAGGTAAAC
**N234**	CAGAGGTGTTGTGGAATTATGGGACTGCTAAGGC
	GCCTTAGCAGTCCCATAATTCCACAACACCTCTG
**N284**	ATGACGTATGGGCCGTATATTACGGGTCACCGC
	GCGGTGACCCGTAATATACGGCCCATACGTCAT
